# Kernel-based distance metric learning for microarray data classification

**DOI:** 10.1186/1471-2105-7-299

**Published:** 2006-06-14

**Authors:** Huilin Xiong, Xue-wen Chen

**Affiliations:** 1 Bioinformatics and Computational Life Sciences Laboratory, Department of Electrical Engineering and Computer Science, University of Kansas, 2335 Irving Hill Road, Lawrence, Kansas 66045, USA; 2 Kansas Masonic Cancer Research Institute, Kansas City, Kansas, USA

## Abstract

**Background:**

The most fundamental task using gene expression data in clinical oncology is to classify tissue samples according to their gene expression levels. Compared with traditional pattern classifications, gene expression-based data classification is typically characterized by high dimensionality and small sample size, which make the task quite challenging.

**Results:**

In this paper, we present a modified K-nearest-neighbor (KNN) scheme, which is based on learning an adaptive distance metric in the data space, for cancer classification using microarray data. The distance metric, derived from the procedure of a data-dependent kernel optimization, can substantially increase the class separability of the data and, consequently, lead to a significant improvement in the performance of the KNN classifier. Intensive experiments show that the performance of the proposed kernel-based KNN scheme is competitive to those of some sophisticated classifiers such as support vector machines (SVMs) and the uncorrelated linear discriminant analysis (ULDA) in classifying the gene expression data.

**Conclusion:**

A novel distance metric is developed and incorporated into the KNN scheme for cancer classification. This metric can substantially increase the class separability of the data in the feature space and, hence, lead to a significant improvement in the performance of the KNN classifier.

## Background

DNA microarray technology is designed to measure the expression levels of tens of thousands of genes simultaneously. As an important application of this novel technology, the gene expression data are used to determine and predict the state of tissue samples, which has shown to be very helpful in clinical oncology. The most fundamental task using gene expression data in clinical oncology is to classify tissue samples according to their gene expression levels. In combination with pattern classification techniques, gene expression data can provide more reliable means to diagnose and predict various types of cancers than the traditional clinical methods.

Compared with traditional pattern classifications, gene expression-based data classification is typically characterized by high dimensionality and small sample size, which make the task quite challenging. In the literature, a number of methods have been applied or developed to classify microarray data [1-6]. These methods include K-nearest-neighbor (KNN), boosting, linear discriminant analysis (LDA), and support vector machines (SVM), etc. we herein briefly review some of the approaches.

### K-Nearest-Neighbor (KNN)

The KNN method is a simple, yet useful approach to data classification. The error rate of the KNN has been proven to be asymptotically at most twice that of the Bayessian error rate [7]. However, its performance deteriorates dramatically when the input data set has a relatively low local relevance [8]. The most important factor impacting the performance of KNN is the distance metric. It is desirable to adopt an appropriate distance metric for the KNN algorithm. In practice, the Euclidean distance is usually used as the distance metric.

### Diagonal Linear Discriminant Analysis (DLDA)

DLDA is the simplest case of the maximum likelihood discriminant rule, in which the class densities are supposed to have the same diagonal covariance matrix. In the special case of binary classification, the DLDA scheme can be viewed as the "weighted voting scheme" proposed by Golub *et al*. in [3]. The major advantage of the DLDA algorithm lies in its computational efficiency.

### Linear Discriminant Analysis (LDA)

The classical LDA method aims to find the most discriminatory projection directions of the input data and classifies the data in the projected space. A major problem in employing the classical LDA algorithm for classifying gene expression data is that the so called scatter matrices are always singular, due to the nature of high dimensionality and relatively small sample size. The singularity makes the classical LDA algorithm inapplicable. In the areas such as face recognition and text classification, the principal component analysis (PCA) technique is introduced as a preprocessing procedure in order to reduce the dimensionality of the input data. However, since the projection criterion of PCA is essentially different from that of LDA, losing discriminatory information in the PCA step becomes inevitable. A recent development in LDA is the generalized discriminant analysis [9,10], in which a more delicate matrix technique, namely, the generalized singular value decomposition (GSVD), is used to modify the classical LDA into a more general version.

### Support Vector Machines (SVM)

SVM has been recognized as the most powerful classifier in various applications of pattern classification. For binary classification, SVM searches for a hyperplane that separates the two classes of data with the maximum margin. It has been shown that support vector machines perform well in many areas of computational biology [11-13]. In the experimental part of this paper, we follow the way in [14] to implement the SVM algorithm.

Generally speaking, due to the high dimensionality and small sample size, linear classifiers such as the linear discrimiant analysis (LDA), and the support vector machines (SVM) with linear kernels are used favorably. However, based on some benchmark tests, researchers have shown that nonlinear classfiers are capable of exploring the nonlinear discriminatory information in the microarray data, and usually produce more precise classification results [15,16]. This is especially true when more patients' samples are available or the data dimension is substantially reduced, since, in these cases, the linear separability of the microarray data could be considerably degraded.

Among the general algorithms of pattern classification, K-nearest-neighbor (KNN) is a simple yet useful one. However, in practice, the performance of KNN algorithm is often inferior to those of the sophisticated approaches such as SVM and generalized linear discriminant analysis (GLDA) [9,10]. Since the distance metric is of great importance for the KNN scheme, an attractive way to improve the performance of KNN is to adopt a more adaptive distance metric to the input data than the Euclidean diatnce. In this paper, we propose to learn the adaptive distance metric via optimizing a data-dependent kernel. Experimental results show that, compared with the ordinary Euclidean distance-based KNN scheme, our kernel-based KNN algorithm, denoted KerNN, always achieves significant improvement in the performance of classifying gene expression data. Moreover, the performance of the KerNN classifier is shown to be competitive, if not better, to those of the sophisticated classifiers, e.g., SVM and the uncorrelated linear discriminant analysis (ULDA) [10], in classifying microarray data.

## Results

We conducted intensive experiments to compare the performances of our KerNN scheme to the commonly-used classification algorithms, i.e., KNN, DLDA [3], ULDA [10], and SVM. Ten publicly available microarray data sets were chosen to test our algorithms. The basic information about these data sets is summarized below. Each data set is first normalized to a distribution with zero mean and unity variance in every feature direction, and then, randomly partitioned into two disjoint subsets with equal number of samples, one is used as the training data, and the other the test data. We only consider Gaussian kernel function in the proposed and SVM algorithms.

1. *ALL-AML Leukemia Data*: This data set, taken from the website [17], contains 72 samples of human acute leukemia. 47 samples belong to acute lymphoblastic leukemia (ALL), and the other acute myeloid leukemia (AML). Each sample presents the expression levels of 7129 genes. For the detailed information, one can refer to [3].

2. *ALL-MLL-AML Leukemia Data*: This leukemia microarray data set is available on the website [17]. It includes 72 human leukemia samples, 24 of them belong to acute lymphoblastic leukemia (ALL), 20 of them to mixed lineage leukemia (MLL), a subset of human acute leukemia with a chromosomal translocation, and 28 of the samples are acute myelogenous leukemia (AML). Each sample gives the expression levels of 12582 genes. Further information about this data set can be found in [21].

3. *Embryonal Tumors of the Central Nervous System (CNS)*: This data set, available at the website [17], contains 60 patient samples, 21 are survivors of a treatment, and 39 are failures. There are 7129 genes in the data set. One can refer to [22] to find more information about this data set.

4. *Breast Cancer Data*: The data are available on the website [18]. The expression matrix monitors 7129 genes in 49 breast tumor samples. There are two response variables respectively describing the status of the estrogen receptor (ER) and the lymph nodal (LN) status. For the ER status, 25 samples are ER+, whereas the remaining 24 samples are ER-. For the LN variable, there are 25 positive sample and 24 negative samples. The detailed information about this data set can be found in [6].

5. *Colon Tumor Data*: This data set is adopted from the website [17]. The data contain 62 samples collected from colon-cancer patients. Among them, 40 samples are from tumors, and 22 normal biopsies are from healthy parts of the colons of the same patients. 2000 genes were selected to measure their expression levels. One can refer to [23].

6. *Lung Cancer Data*: This data set is taken from the website [17]. It contains 181 tissue samples, which are classified into two classes: malignant pleural mesothelioma (MPM) and adenocarcinoma (ADCA). Each sample is described by 12533 genes. More information about this data set can be found in [24].

7. *Lymphoma Data*: The data are available on the website [19]. This data set contains 77 tissue samples, 58 are diffuse large B-cell lymphomas (DLBCL) and the remaining 19 samples are follicular lymphomas (FL). Each sample is represented by the expression levels of 7129 genes. The detailed information about this data set can be found in [25].

8. *Ovarian Cancer Data*: This data set, available on the website [17], is to distinguish ovarian cancer from non-cancer. It contains 253 samples, and each sample has 15154 features. More details can be found in [26].

9. *Prostate Cancer Data*: This data set, adopted from the website [19], contains the gene expression levels of 12600 genes for 52 prostate tumor samples and 50 normal prostate samples. One can refer [4] for the details about this data set.

10. *Subtypes of Acute Lymphoblastic Leukemia*: This data set, available on the website [20], contains 6 subtypes of pediatric acute lymphoblastic leukemia, corresponding to six diagnostic groups: BCR-ABL, E2A-PBX1, MLL, T-ALL, TEL-AML1, Hyperdiloid>50. Each sample contains 12625 genes.

### Comparisons in terms of the best results

For each data set, we chose the *N*_*f *_most discriminatory genes, where *N*_*f *_= 10, 20, 40, 60, 80, 100, 200, 400, 600, 800, 1000, 1200, 1400, 1600, 1800, 2000, respectively; repeated the experiment 100 times at each value of *N*_*f*_; and then, calculated the average test error rates and their standard deviations over the 100 experiments. Table 1 lists the best results, i.e., the smallest average test error rate, of different algorithms. It can be seen that the proposed KerNN algorithm reaches the best, which are in bold face, on four data sets. On the other data sets, the performance of the KerNN algorithm is still competitive, if not better, to those of the SVM and ULDA schemes.

In Table 1, if we assign a score 1 to the best result, 2 to the next best result, ..., and so on, then, the global performance of a classifier can be roughly evaluated in terms of the average score. We show the average scores of the five classifiers in Table 1. It can be seen that the proposed KerNN scheme achieves the lowest score among the five classifiers.

### Comparisons under different gene numbers

**Figure 1:**
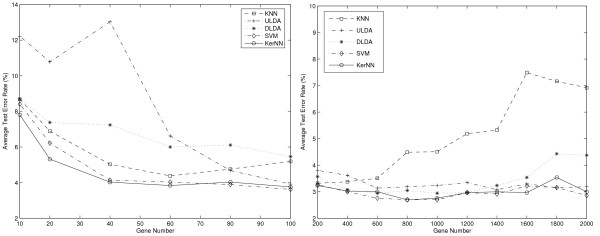
**Test error rates for the ALL-AML data set**. Stability comparison on the *ALL-AML *data. The average test error rate (%) as a function of the selected gene number.

**Figure 2:**
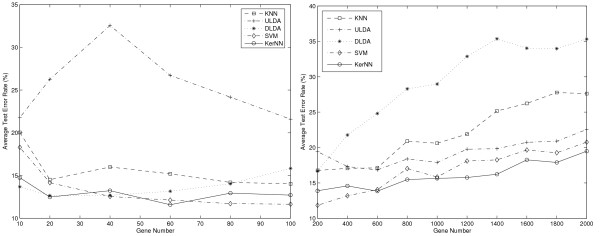
**Test error rates for the Colon data set**. Stability comparison on the *Colon *data. The average test error rate (%) as a function of the selected gene number.

**Figure 3:**
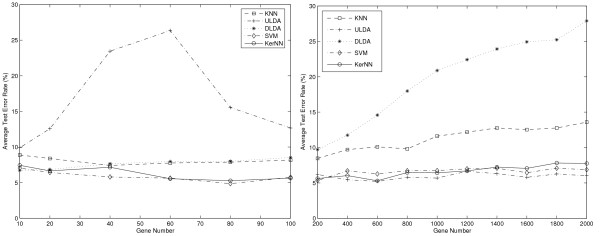
**Test error rates for the Prostate data set**. Stability comparison on the *Prostate *data. The average test error rate (%) as a function of the selected gene number.

To investigate the stability of the 5 classification algorithms, we compared their performances when different number of genes were selected. The experimental results are shown in Fig. 1, for the *ALL-AML *data, Fig. 2, for the *Colon *data, and Fig. 3, for the *Prostate *data, where the horizontal axis is the number of the selected genes and the vertical axis is the average test error rates of the classifiers over 100 experiments. While Fig. 1 (a), Fig. 2 (a), and Fig. 3 (a) illustrate the results in the case of choosing a relatively small number of features (from 10 to 100), Fig. 1 (b), Fig. 2 (b), and Fig. 3 (b) demonstrate the corresponding results when more genes are chosen (from 200 to 2000). It can be seen that the proposed KerNN scheme performs favorably in most cases. Compared with the ULDA scheme, which always performs poorly in the case of small feature size, and the DLDA algorithm, whose performances usually degrade for relatively large feature size, our KerNN algorithm works with more stability with different feature numbers. More importantly, compared with the ordinary KNN classifier, the kernel optimization-based KNN classifier always gains significant improvements, which implies that the procedure of kernel optimization induces a distance metric that adapts better than the Euclidean metric to the gene expression data in the data space.

## Discussion

### Parameter tuning

In the experiments, for KNN, ULDA, and the proposed algorithm, the final classification is done via the K-nearest-neighbor algorithm with K = 3. For KNN, ULDA, and DLDA algorithms, the only parameter is the number of selected genes *N*_*f*_. For SVM, in addition to the gene number, two parameters, the *γ *in the Gaussian kernel function and the regulation constant *C*, need to be set in advance. As for the KerNN algorithm, there are more parameters. To avoid the intensive computation in parameter tuning using the cross validation, we respectively chose the *N*_*f *_most discriminatory genes, where *N*_*f *_= 10, 20, 40, 60, 80, 100, 200, 400, 600, 800, 1000, 1200, 1400, 1600, 1800, 2000. The best performance for each method is reported in Table 1. For our kernel optimization method, the initial learning rate *η*_0 _and the total iteration number *N *are always set to 0.01 and 1000 respectively. Furthermore, for the sake of computational simplicity, we empirically set the two Gaussian parameters in the proposed method as γ0=10−5Nf
 MathType@MTEF@5@5@+=feaafiart1ev1aaatCvAUfKttLearuWrP9MDH5MBPbIqV92AaeXatLxBI9gBaebbnrfifHhDYfgasaacH8akY=wiFfYdH8Gipec8Eeeu0xXdbba9frFj0=OqFfea0dXdd9vqai=hGuQ8kuc9pgc9s8qqaq=dirpe0xb9q8qiLsFr0=vr0=vr0dc8meaabaqaciaacaGaaeqabaqabeGadaaakeaaiiGacqWFZoWzdaWgaaWcbaGaeGimaaJaeyypa0dabeaakmaalaaabaGaeGymaeJaeGimaaZaaWbaaSqabeaacqGHsislcqaI1aqnaaaakeaadaGcaaqaaiabd6eaonaaBaaaleaacqWGMbGzaeqaaaqabaaaaaaa@3744@ and γ1=10−2Nf
 MathType@MTEF@5@5@+=feaafiart1ev1aaatCvAUfKttLearuWrP9MDH5MBPbIqV92AaeXatLxBI9gBaebbnrfifHhDYfgasaacH8akY=wiFfYdH8Gipec8Eeeu0xXdbba9frFj0=OqFfea0dXdd9vqai=hGuQ8kuc9pgc9s8qqaq=dirpe0xb9q8qiLsFr0=vr0=vr0dc8meaabaqaciaacaGaaeqabaqabeGadaaakeaaiiGacqWFZoWzdaWgaaWcbaGaeGymaedabeaakiabg2da9maalaaabaGaeGymaeJaeGimaaZaaWbaaSqabeaacqGHsislcqaIYaGmaaaakeaadaGcaaqaaiabd6eaonaaBaaaleaacqWGMbGzaeqaaaqabaaaaaaa@3740@ , rather than tune them by the cross validation. This may not be the optimal settings for the parameters *γ*_0 _and *γ*_1_. However, high computational complexity can be avoided. It is expected that even better results could be obtained if we were to choose them by the cross validation. Therefore, for the KerNN method, there is only one parameter *σ*_*ε*_, the standard variance of the disturbance added to the data in Eq. (10), that need to be tuned. As to the SVM, two parameters are tuned by the cross validation.

In the experiments, we employed the leave-one-out technique on the training data to choose these parameters. We followed [14] to implement the SVM algorithm, in which the parameter *C *is chosen from {l.0e+00, l.0e+01, l.0e+02, l.0e+03, l.0e+04, l.0e+05, l.0e+06, l.0e+07} and *γ *from {l.0e-07, 5.0e-07, l.0e-06, 5.0e-06, l.0e-05, 5.0e-05, l.0e-04, 5.0e-04, l.0e-03, 5.0e-03, l.0e-02} using the leave-one-out cross validation. For our KerNN algorithm, the parameter *σ*_*ε *_is selected from {0, 0.1, 0.2, 0.3, 0.4, 0.5, 0.6, 0.7, 0.8, 0.9, 1.0}. Note that only the training samples were used for setting parameters. Test samples are independent of this process.

### Gene selection

In this paper, we employ the BW ratio used in [2,10] to select genes. This ratio is essentially a Fisher discriminant measure. Given a gene *j*, the ratio on gene *j *is calculated as

g(j)=∑k=1pmk(x¯k(j)−x¯(j))2∑k=1p∑i∈Ck(xi(j)−x¯k(j))2
 MathType@MTEF@5@5@+=feaafiart1ev1aaatCvAUfKttLearuWrP9MDH5MBPbIqV92AaeXatLxBI9gBaebbnrfifHhDYfgasaacH8akY=wiFfYdH8Gipec8Eeeu0xXdbba9frFj0=OqFfea0dXdd9vqai=hGuQ8kuc9pgc9s8qqaq=dirpe0xb9q8qiLsFr0=vr0=vr0dc8meaabaqaciaacaGaaeqabaqabeGadaaakeaacqWGNbWzcqGGOaakcqWGQbGAcqGGPaqkcqGH9aqpdaWcaaqaamaaqadabaGaemyBa02aaSbaaSqaaiabdUgaRbqabaGccqGGOaakcuWG4baEgaqeamaaBaaaleaacqWGRbWAaeqaaOGaeiikaGIaemOAaOMaeiykaKIaeyOeI0IafmiEaGNbaebacqGGOaakcqWGQbGAcqGGPaqkcqGGPaqkdaahaaWcbeqaaiabikdaYaaaaeaacqWGRbWAcqGH9aqpcqaIXaqmaeaacqWGWbaCa0GaeyyeIuoaaOqaamaaqadabaWaaabeaeaacqGGOaakcqWG4baEdaWgaaWcbaGaemyAaKgabeaakiabcIcaOiabdQgaQjabcMcaPiabgkHiTiqbdIha4zaaraWaaSbaaSqaaiabdUgaRbqabaGccqGGOaakcqWGQbGAcqGGPaqkcqGGPaqkdaahaaWcbeqaaiabikdaYaaaaeaacqWGPbqAcqGHiiIZcqWGdbWqdaWgaaadbaGaem4AaSgabeaaaSqab0GaeyyeIuoaaSqaaiabdUgaRjabg2da9iabigdaXaqaaiabdchaWbqdcqGHris5aaaaaaa@689B@

where *C*_*k *_denotes the index set of the *k*-th class (*k *= l,2,...,*p*), *m*_*k *_is the number of samples in *C*_*k *_(∑k=1pmk=m
 MathType@MTEF@5@5@+=feaafiart1ev1aaatCvAUfKttLearuWrP9MDH5MBPbIqV92AaeXatLxBI9gBaebbnrfifHhDYfgasaacH8akY=wiFfYdH8Gipec8Eeeu0xXdbba9frFj0=OqFfea0dXdd9vqai=hGuQ8kuc9pgc9s8qqaq=dirpe0xb9q8qiLsFr0=vr0=vr0dc8meaabaqaciaacaGaaeqabaqabeGadaaakeaadaaeWaqaaiabd2gaTnaaBaaaleaacqWGRbWAaeqaaaqaaiabdUgaRjabg2da9iabigdaXaqaaiabdchaWbqdcqGHris5aOGaeyypa0JaemyBa0gaaa@38C2@), x¯
 MathType@MTEF@5@5@+=feaafiart1ev1aaatCvAUfKttLearuWrP9MDH5MBPbIqV92AaeXatLxBI9gBaebbnrfifHhDYfgasaacH8akY=wiFfYdH8Gipec8Eeeu0xXdbba9frFj0=OqFfea0dXdd9vqai=hGuQ8kuc9pgc9s8qqaq=dirpe0xb9q8qiLsFr0=vr0=vr0dc8meaabaqaciaacaGaaeqabaqabeGadaaakeaacuWG4baEgaqeaaaa@2E3D@_*k*_(*j*) and x¯
 MathType@MTEF@5@5@+=feaafiart1ev1aaatCvAUfKttLearuWrP9MDH5MBPbIqV92AaeXatLxBI9gBaebbnrfifHhDYfgasaacH8akY=wiFfYdH8Gipec8Eeeu0xXdbba9frFj0=OqFfea0dXdd9vqai=hGuQ8kuc9pgc9s8qqaq=dirpe0xb9q8qiLsFr0=vr0=vr0dc8meaabaqaciaacaGaaeqabaqabeGadaaakeaacuWG4baEgaqeaaaa@2E3D@(*j*) represent the average expression levels cross the *k*-th class and whole training samples on gene *j*, respectively.

Gene selection usually has a strong impact on the performances of various classifiers, due to the effect of correlation between genes. Our experiments show that the impact can be considered in two aspects: l)with different numbers of genes, the performance of a classifier could be remarkably different. For example, the ULDA method usually works quite well as a large number of genes is used, but performs poorly in the case of small gene number. Contrarily, the DLDA classifier often reaches its best performance at small number of features. 2) with different numbers of genes, the model parameters, especially for the nonlinear methods, need to be set differently to achieve better result.

### The effect of the disturbed resampling

Due to the lack of enough training samples, the scheme of the kernel optimization-based classification may lead to an overfitting result in classifying gene expression data. To alleviate the possible overfitting, a strategy of disturbed resampling, as shown in Eq. (10), was adopted. In this section, we demonstrate that using this strategy, the overfiting could be effectively reduced.

**Figure 4:**
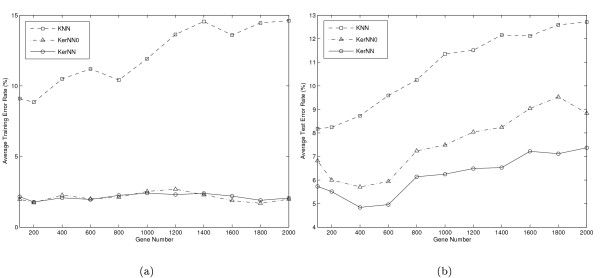
**The effect of the disturbed resampling on Prostate**. The effect of adopting the technique of disturbed resampling on a relatively large data set, *Prostate*, which contains 102 samples. (a) Results on the training data. (b) Results on the test data.

**Figure 5:**
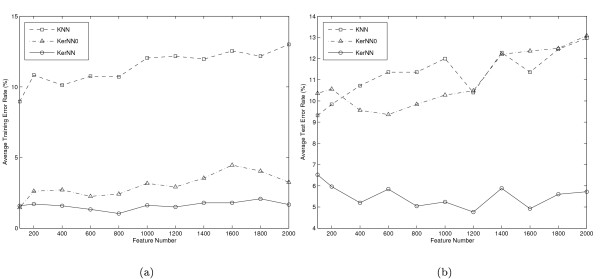
**The effect of the disturbed resampling on Breast-ER**. The effect of adopting the technique of disturbed resampling on the a relatively small data set, *Breast-ER*, which contains only 49 samples. (a) Results on the training data. (b) Results on the test data.

In the case that there are relatively large number of samples, the kernel optimization-based KNN classifier without using the strategy of disturbed resampling, denoted by KerNN0, usually works well on both the training and test data. Fig. 4 illustrates the performances of KNN, KerNN0, and KerNN on both the training and test data of the *Prostate *data set, which includes 102 samples. It can be seen that, compared with the KNN algorithm, both the KerNN0 and KerNN methods gain significant improvements, not only on the training data, but also on the test data. However, when the sample size is relatively small, the KerNNO algorithm may lead to serious overfitting. We choose the *Breast-ER *data set, which contains only 49 samples, to demonstrate our argument. Fig. 5 (a) shows the average error rates of KNN, KerNN0, and KerNN algorithms on the training data, and Fig. 5 (b) presents the corresponding results on the test data. It can be seen that, although KerNN0 works quite well on the training data, its performance degrades remarkably on the test data. On the contrary, for the KerNN scheme, no overfitting occurred.

## Conclusion

In this paper, a novel distance metric is developed and incorporated into a KNN scheme for cancer classification. This metric, derived from the procedure of a data-dependent kernel optimization, can substantially increase the class separability of the data in the feature space, and hence, lead to a significant improvement in the performance of the KNN classifier. Furthermore, in combination with a disturbed resampling strategy, the kernel optimization-based K-nearest-neighbor scheme can achieve competitive performance to the fine tuned SVM and the uncorrelated linear discriminant analysis (ULDA) scheme in classifying gene expression data. Experimental results show that the proposed scheme performs with more stability than the ULDA scheme, which works poorly in the case of small feature size, and the DLDA scheme, whose performance usually degrades in the case of a relatively large feature size.

## Methods

### 0.1 Data-dependent kernel model

In this paper, we employ a special kernel function model, which is called date-dependent kernel model, as the objective kernel to be optimized. Apparently, there is no benefit at all if we simply use the common kernel such as the Gaussian kernel or the polynomial kernel in the KNN scheme, since the distance ranking in the Hilbert space derived from the kernel function is the same as that in the input data space. However, when we adopt the data-dependent kernel, especially after the kernel is optimized, the distance metric could be appropriately modified so that the local relevance of the data is significantly improved.

Let {*x*_*i*_, *ζ*_*i*_} (*i *= 1,2, ..., *m*) be *m d*-dimensional training samples of the given gene expression data, where *ζ*_*i *_represent the class labels of the samples. We refer the data-dependent kernel as,

*k*(*x*, *y*) = *q*(*x*)*q*(*y*)*k*_0_(*x*, *y*)       (1)

where *x*, *y *∈ **R**^*d*^, *k*_0_(*x*, *y*), called the basic kernel, is an ordinary kernel such as a Gaussian or a polynomial kernel function, and *q*(.), the factor function, takes the form as

q(x)=α0+∑i=1lαik1(x,ai)     (2)
 MathType@MTEF@5@5@+=feaafiart1ev1aaatCvAUfKttLearuWrP9MDH5MBPbIqV92AaeXatLxBI9gBaebbnrfifHhDYfgasaacH8akY=wiFfYdH8Gipec8Eeeu0xXdbba9frFj0=OqFfea0dXdd9vqai=hGuQ8kuc9pgc9s8qqaq=dirpe0xb9q8qiLsFr0=vr0=vr0dc8meaabaqaciaacaGaaeqabaqabeGadaaakeaacqWGXbqCcqGGOaakcqWG4baEcqGGPaqkcqGH9aqpiiGacqWFXoqydaWgaaWcbaGaeGimaadabeaakiabgUcaRmaaqahabaGae8xSde2aaSbaaSqaaiabdMgaPbqabaGccqWGRbWAdaWgaaWcbaGaeGymaedabeaakiabcIcaOiabdIha4jabcYcaSiabdggaHnaaBaaaleaacqWGPbqAaeqaaOGaeiykaKcaleaacqWGPbqAcqGH9aqpcqaIXaqmaeaacqWGSbaBa0GaeyyeIuoakiaaxMaacaWLjaWaaeWaaeaacqaIYaGmaiaawIcacaGLPaaaaaa@4D48@

in which *k*_1_(*x*, *a*_*i*_) = e−γ1||x−ai||2
 MathType@MTEF@5@5@+=feaafiart1ev1aaatCvAUfKttLearuWrP9MDH5MBPbIqV92AaeXatLxBI9gBaebbnrfifHhDYfgasaacH8akY=wiFfYdH8Gipec8Eeeu0xXdbba9frFj0=OqFfea0dXdd9vqai=hGuQ8kuc9pgc9s8qqaq=dirpe0xb9q8qiLsFr0=vr0=vr0dc8meaabaqaciaacaGaaeqabaqabeGadaaakeaacqWGLbqzdaahaaWcbeqaaiabgkHiTGGaciab=n7aNnaaBaaameaacqaIXaqmaeqaaSGaeiiFaWNaeiiFaWNaemiEaGNaeyOeI0Iaemyyae2aaSbaaWqaaiabdMgaPbqabaWccqGG8baFcqGG8baFdaahaaadbeqaaiabikdaYaaaaaaaaa@3E53@, *α*_*i*_'s are the combination coefficients, and *a*_*i*_'s denote the local centers of the training data.

Let the kernel matrices corresponding to *k*(*x*, *y*) and *k*_0_(*x*, *y*) be *K *and *k*_0_. Obviously, *K *= [*q*(*x*_*i*_)*q*(*x*_*j*_)*k*_0_(*x*_*i*_, *x*_*j*_)]_*m *× *m *_= *QK*_0_*Q*, where *Q *is a diagonal matrix whose diagonal elements are *q*(*x*_1_), *q*(*x*_2_),...,*q*(*x*_*m*_). Let us denote the vector (*q*(*x*_1_), *q*(*x*_2_),..., *q*(*x*_*m*_))^*T *^and (*α*_0_, *α*_1_, *α*_2_,...,*α*_*n*_)^*T *^by *q *and *α *respectively, we have *q *= *K*_1_*α*, where *K*_1 _is an *m *× (*l *+ 1) matrix

K1=(1k1(x1,a1)⋯k1(x1,al)1k1(x2,a1)⋯k1(x2,al)⋮⋮⋱⋮1k1(xm,a1)⋯k1(xm,al))     (3)
 MathType@MTEF@5@5@+=feaafiart1ev1aaatCvAUfKttLearuWrP9MDH5MBPbIqV92AaeXatLxBI9gBaebbnrfifHhDYfgasaacH8akY=wiFfYdH8Gipec8Eeeu0xXdbba9frFj0=OqFfea0dXdd9vqai=hGuQ8kuc9pgc9s8qqaq=dirpe0xb9q8qiLsFr0=vr0=vr0dc8meaabaqaciaacaGaaeqabaqabeGadaaakeaacqWGlbWsdaWgaaWcbaGaeGymaedabeaakiabg2da9maabmaabaqbaeqabqabaaaaaeaacqaIXaqmaeaacqWGRbWAdaWgaaWcbaGaeGymaedabeaakiabcIcaOiabdIha4naaBaaaleaacqaIXaqmaeqaaOGaeiilaWIaemyyae2aaSbaaSqaaiabigdaXaqabaGccqGGPaqkaeaacqWIVlctaeaacqWGRbWAdaWgaaWcbaGaeGymaedabeaakiabcIcaOiabdIha4naaBaaaleaacqaIXaqmaeqaaOGaeiilaWIaemyyae2aaSbaaSqaaiabdYgaSbqabaGccqGGPaqkaeaacqaIXaqmaeaacqWGRbWAdaWgaaWcbaGaeGymaedabeaakiabcIcaOiabdIha4naaBaaaleaacqaIYaGmaeqaaOGaeiilaWIaemyyae2aaSbaaSqaaiabigdaXaqabaGccqGGPaqkaeaacqWIVlctaeaacqWGRbWAdaWgaaWcbaGaeGymaedabeaakiabcIcaOiabdIha4naaBaaaleaacqaIYaGmaeqaaOGaeiilaWIaemyyae2aaSbaaSqaaiabdYgaSbqabaGccqGGPaqkaeaacqWIUlstaeaacqWIUlstaeaacqWIXlYtaeaacqWIUlstaeaacqaIXaqmaeaacqWGRbWAdaWgaaWcbaGaeGymaedabeaakiabcIcaOiabdIha4naaBaaaleaacqWGTbqBaeqaaOGaeiilaWIaemyyae2aaSbaaSqaaiabigdaXaqabaGccqGGPaqkaeaacqWIVlctaeaacqWGRbWAdaWgaaWcbaGaeGymaedabeaakiabcIcaOiabdIha4naaBaaaleaacqWGTbqBaeqaaOGaeiilaWIaemyyae2aaSbaaSqaaiabdYgaSbqabaGccqGGPaqkaaaacaGLOaGaayzkaaGaaCzcaiaaxMaadaqadaqaaiabiodaZaGaayjkaiaawMcaaaaa@84DF@

### 0.2 Kernel optimization for binary-class data

We optimized the data-dependent kernel in Eq.(l). This requires optimizing the combination coefficient vector *α*, aiming to increase the class separability of the data in the feature space. A Fisher scalar measuring the class separability of the training data in the feature space is adopted as a criterion for our kernel optimization

J=tr(Sb)tr(Sw)     (4)
 MathType@MTEF@5@5@+=feaafiart1ev1aaatCvAUfKttLearuWrP9MDH5MBPbIqV92AaeXatLxBI9gBaebbnrfifHhDYfgasaacH8akY=wiFfYdH8Gipec8Eeeu0xXdbba9frFj0=OqFfea0dXdd9vqai=hGuQ8kuc9pgc9s8qqaq=dirpe0xb9q8qiLsFr0=vr0=vr0dc8meaabaqaciaacaGaaeqabaqabeGadaaakeaacqWGkbGscqGH9aqpdaWcaaqaaiabbsha0jabbkhaYjabcIcaOiabdofatnaaBaaaleaacqWGIbGyaeqaaOGaeiykaKcabaGaeeiDaqNaeeOCaiNaeiikaGIaem4uam1aaSbaaSqaaiabdEha3bqabaGccqGGPaqkaaGaaCzcaiaaxMaadaqadaqaaiabisda0aGaayjkaiaawMcaaaaa@4148@

where *S*_*b *_represents the "between-class scatter matrix", and *S*_*w *_"within-class scatter matrix".

Suppose that the training data are grouped according to their class labels, i.e., the first *m*_1 _data belong to one class, and the remaining *m*_2 _data belong to the other class (*m*_1 _+ *m*_2 _= *m*). Then the basic kernel matrix *k*_0 _can be partitioned as

K0=(K110K120K210K220)     (5)
 MathType@MTEF@5@5@+=feaafiart1ev1aaatCvAUfKttLearuWrP9MDH5MBPbIqV92AaeXatLxBI9gBaebbnrfifHhDYfgasaacH8akY=wiFfYdH8Gipec8Eeeu0xXdbba9frFj0=OqFfea0dXdd9vqai=hGuQ8kuc9pgc9s8qqaq=dirpe0xb9q8qiLsFr0=vr0=vr0dc8meaabaqaciaacaGaaeqabaqabeGadaaakeaacqWGlbWsdaWgaaWcbaGaeGimaadabeaakiabg2da9maabmaabaqbaeqabiGaaaqaaiabdUealnaaDaaaleaacqaIXaqmcqaIXaqmaeaacqaIWaamaaaakeaacqWGlbWsdaqhaaWcbaGaeGymaeJaeGOmaidabaGaeGimaadaaaGcbaGaem4saS0aa0baaSqaaiabikdaYiabigdaXaqaaiabicdaWaaaaOqaaiabdUealnaaDaaaleaacqaIYaGmcqaIYaGmaeaacqaIWaamaaaaaaGccaGLOaGaayzkaaGaaCzcaiaaxMaadaqadaqaaiabiwda1aGaayjkaiaawMcaaaaa@45EB@

where the sizes of the submatrices K110,K120,K210
 MathType@MTEF@5@5@+=feaafiart1ev1aaatCvAUfKttLearuWrP9MDH5MBPbIqV92AaeXatLxBI9gBaebbnrfifHhDYfgasaacH8akY=wiFfYdH8Gipec8Eeeu0xXdbba9frFj0=OqFfea0dXdd9vqai=hGuQ8kuc9pgc9s8qqaq=dirpe0xb9q8qiLsFr0=vr0=vr0dc8meaabaqaciaacaGaaeqabaqabeGadaaakeaacqWGlbWsdaqhaaWcbaGaeGymaeJaeGymaedabaGaeGimaadaaOGaeiilaWIaem4saS0aa0baaSqaaiabigdaXiabikdaYaqaaiabicdaWaaakiabcYcaSiabdUealnaaDaaaleaacqaIYaGmcqaIXaqmaeaacqaIWaamaaaaaa@3AD2@, and K220
 MathType@MTEF@5@5@+=feaafiart1ev1aaatCvAUfKttLearuWrP9MDH5MBPbIqV92AaeXatLxBI9gBaebbnrfifHhDYfgasaacH8akY=wiFfYdH8Gipec8Eeeu0xXdbba9frFj0=OqFfea0dXdd9vqai=hGuQ8kuc9pgc9s8qqaq=dirpe0xb9q8qiLsFr0=vr0=vr0dc8meaabaqaciaacaGaaeqabaqabeGadaaakeaacqWGlbWsdaqhaaWcbaGaeGOmaiJaeGOmaidabaGaeGimaadaaaaa@30CA@ respectively are *m*_1 _× *m*_1_, *m*_1 _× *m*_2_, *m*_2 _× *m*_1_, and *m*_2 _× *m*_2_. A close relation between the class separability measure *J *and the kernel matrices can be established [27].

J(α)=αTM0ααTN0α     (6)
 MathType@MTEF@5@5@+=feaafiart1ev1aaatCvAUfKttLearuWrP9MDH5MBPbIqV92AaeXatLxBI9gBaebbnrfifHhDYfgasaacH8akY=wiFfYdH8Gipec8Eeeu0xXdbba9frFj0=OqFfea0dXdd9vqai=hGuQ8kuc9pgc9s8qqaq=dirpe0xb9q8qiLsFr0=vr0=vr0dc8meaabaqaciaacaGaaeqabaqabeGadaaakeaacqWGkbGscqGGOaakiiGacqWFXoqycqGGPaqkcqGH9aqpdaWcaaqaaiab=f7aHnaaCaaaleqabaGaemivaqfaaOGaemyta00aaSbaaSqaaiabicdaWaqabaGccqWFXoqyaeaacqWFXoqydaahaaWcbeqaaiabdsfaubaakiabd6eaonaaBaaaleaacqaIWaamaeqaaOGae8xSdegaaiaaxMaacaWLjaWaaeWaaeaacqaI2aGnaiaawIcacaGLPaaaaaa@43C6@

where *M*_0 _= K1T
 MathType@MTEF@5@5@+=feaafiart1ev1aaatCvAUfKttLearuWrP9MDH5MBPbIqV92AaeXatLxBI9gBaebbnrfifHhDYfgasaacH8akY=wiFfYdH8Gipec8Eeeu0xXdbba9frFj0=OqFfea0dXdd9vqai=hGuQ8kuc9pgc9s8qqaq=dirpe0xb9q8qiLsFr0=vr0=vr0dc8meaabaqaciaacaGaaeqabaqabeGadaaakeaacqWGlbWsdaqhaaWcbaGaeGymaedabaGaemivaqfaaaaa@3019@*B*_0_*K*_1_, *N*_0 _= K1T
 MathType@MTEF@5@5@+=feaafiart1ev1aaatCvAUfKttLearuWrP9MDH5MBPbIqV92AaeXatLxBI9gBaebbnrfifHhDYfgasaacH8akY=wiFfYdH8Gipec8Eeeu0xXdbba9frFj0=OqFfea0dXdd9vqai=hGuQ8kuc9pgc9s8qqaq=dirpe0xb9q8qiLsFr0=vr0=vr0dc8meaabaqaciaacaGaaeqabaqabeGadaaakeaacqWGlbWsdaqhaaWcbaGaeGymaedabaGaemivaqfaaaaa@3019@*W*_0_*K*_1_, in which

B0=(1m1K110001m2K220)−1mK0
 MathType@MTEF@5@5@+=feaafiart1ev1aaatCvAUfKttLearuWrP9MDH5MBPbIqV92AaeXatLxBI9gBaebbnrfifHhDYfgasaacH8akY=wiFfYdH8Gipec8Eeeu0xXdbba9frFj0=OqFfea0dXdd9vqai=hGuQ8kuc9pgc9s8qqaq=dirpe0xb9q8qiLsFr0=vr0=vr0dc8meaabaqaciaacaGaaeqabaqabeGadaaakeaacqWGcbGqdaWgaaWcbaGaeGimaadabeaakiabg2da9maabmaabaqbaeqabiGaaaqaamaalaaabaGaeGymaedabaGaemyBa02aaSbaaSqaaiabigdaXaqabaaaaOGaem4saS0aa0baaSqaaiabigdaXiabigdaXaqaaiabicdaWaaaaOqaaiabicdaWaqaaiabicdaWaqaamaalaaabaGaeGymaedabaGaemyBa02aaSbaaSqaaiabikdaYaqabaaaaOGaem4saS0aa0baaSqaaiabikdaYiabikdaYaqaaiabicdaWaaaaaaakiaawIcacaGLPaaacqGHsisldaWcaaqaaiabigdaXaqaaiabd2gaTbaacqWGlbWsdaWgaaWcbaGaeGimaadabeaaaaa@4841@

W0=diag(k110,k220,…,kmm0)−(1m1K110001m2K220)
 MathType@MTEF@5@5@+=feaafiart1ev1aaatCvAUfKttLearuWrP9MDH5MBPbIqV92AaeXatLxBI9gBaebbnrfifHhDYfgasaacH8akY=wiFfYdH8Gipec8Eeeu0xXdbba9frFj0=OqFfea0dXdd9vqai=hGuQ8kuc9pgc9s8qqaq=dirpe0xb9q8qiLsFr0=vr0=vr0dc8meaabaqaciaacaGaaeqabaqabeGadaaakeaacqWGxbWvdaWgaaWcbaGaeGimaadabeaakiabg2da9iabbsgaKjabbMgaPjabbggaHjabbEgaNnaabmaabaGaem4AaS2aa0baaSqaaiabigdaXiabigdaXaqaaiabicdaWaaakiabcYcaSiabdUgaRnaaDaaaleaacqaIYaGmcqaIYaGmaeaacqaIWaamaaGccqGGSaalcqWIMaYscqGGSaalcqWGRbWAdaqhaaWcbaGaemyBa0MaemyBa0gabaGaeGimaadaaaGccaGLOaGaayzkaaGaeyOeI0YaaeWaaeaafaqabeGacaaabaWaaSaaaeaacqaIXaqmaeaacqWGTbqBdaWgaaWcbaGaeGymaedabeaaaaGccqWGlbWsdaqhaaWcbaGaeGymaeJaeGymaedabaGaeGimaadaaaGcbaGaeGimaadabaGaeGimaadabaWaaSaaaeaacqaIXaqmaeaacqWGTbqBdaWgaaWcbaGaeGOmaidabeaaaaGccqWGlbWsdaqhaaWcbaGaeGOmaiJaeGOmaidabaGaeGimaadaaaaaaOGaayjkaiaawMcaaaaa@5C76@

To avoid using the eigenvector solution, an updating algorithm based on the standard gradient approach is developed. This algorithm is summarized below, in which the learning rate *η*(*n*) is adopted in a gradually decreasing form as

η(n)=η0(1−nN)     (7)
 MathType@MTEF@5@5@+=feaafiart1ev1aaatCvAUfKttLearuWrP9MDH5MBPbIqV92AaeXatLxBI9gBaebbnrfifHhDYfgasaacH8akY=wiFfYdH8Gipec8Eeeu0xXdbba9frFj0=OqFfea0dXdd9vqai=hGuQ8kuc9pgc9s8qqaq=dirpe0xb9q8qiLsFr0=vr0=vr0dc8meaabaqaciaacaGaaeqabaqabeGadaaakeaaiiGacqWF3oaAcqGGOaakcqWGUbGBcqGGPaqkcqGH9aqpcqWF3oaAdaWgaaWcbaGaeGimaadabeaakiabcIcaOiabigdaXiabgkHiTmaalaaabaGaemOBa4gabaGaemOta4eaaiabcMcaPiaaxMaacaWLjaWaaeWaaeaacqaI3aWnaiaawIcacaGLPaaaaaa@3F39@

where *η*_0 _represents an initial learning rate.

1. Group the data according to their class labels. Calculate *K*_0 _and *K*_1 _first, then *B*_0 _and *W*_0_, and then *M*_0_, *N*_0_.

2. Initialize *α*^(0) ^by a vector (1,0,..., 0)^*T*^, and set *n *= 0.

3. Calculate *q *= *K*_1_*α*^(*n*)^, and *J*_1 _= *q*^*T *^*B*_0_*q*, *J*_2 _= *q*^*T *^*W*_0_*q*, and *J*.

4. Update *α*^(*n*)^:

α(n+1)=α(n)+η(n)(1J2M0−1J2N0)α(n)
 MathType@MTEF@5@5@+=feaafiart1ev1aaatCvAUfKttLearuWrP9MDH5MBPbIqV92AaeXatLxBI9gBaebbnrfifHhDYfgasaacH8akY=wiFfYdH8Gipec8Eeeu0xXdbba9frFj0=OqFfea0dXdd9vqai=hGuQ8kuc9pgc9s8qqaq=dirpe0xb9q8qiLsFr0=vr0=vr0dc8meaabaqaciaacaGaaeqabaqabeGadaaakeaaiiGacqWFXoqydaahaaWcbeqaaiabcIcaOiabd6gaUjabgUcaRiabigdaXiabcMcaPaaakiabg2da9iab=f7aHnaaCaaaleqabaGaeiikaGIaemOBa4MaeiykaKcaaOGaey4kaSIae83TdGMaeiikaGIaemOBa4MaeiykaKIaeiikaGYaaSaaaeaacqaIXaqmaeaacqWGkbGsdaWgaaWcbaGaeGOmaidabeaaaaGccqWGnbqtdaWgaaWcbaGaeGimaadabeaakiabgkHiTmaalaaabaGaeGymaedabaGaemOsaO0aaSbaaSqaaiabikdaYaqabaaaaOGaemOta40aaSbaaSqaaiabicdaWaqabaGccqGGPaqkcqWFXoqydaahaaWcbeqaaiabcIcaOiabd6gaUjabcMcaPaaaaaa@5197@

and normalize *α*^(*n*+1) ^so that ||*α*^(*n*+1)^|| = 1.

5. If *n *reaches a pre-specified number *N*, stop. Otherwise, set *n *= *n *+ 1, go to 3.

### 0.3 Kernel optimization for multi-class data

In the case of multi-class data, we decompose the problem of kernel optimization into a series of binary-class kernel optimizations.

Let (*x*_*i*_, *ζ*_*i*_) ∈ **R**^*d *^× *ζ *(*i *= 1, 2,..., *m*) be the training data set containing *p *classes, that is, *ζ *= {1,2,...,*p*}. We assume the data to be grouped in order, that is, the first *m*_1 _data belong to the first class, the next *m*_2 _data belong to the second class, and so on, where ∑i=1pmi=m
 MathType@MTEF@5@5@+=feaafiart1ev1aaatCvAUfKttLearuWrP9MDH5MBPbIqV92AaeXatLxBI9gBaebbnrfifHhDYfgasaacH8akY=wiFfYdH8Gipec8Eeeu0xXdbba9frFj0=OqFfea0dXdd9vqai=hGuQ8kuc9pgc9s8qqaq=dirpe0xb9q8qiLsFr0=vr0=vr0dc8meaabaqaciaacaGaaeqabaqabeGadaaakeaadaaeWaqaaiabd2gaTnaaBaaaleaacqWGPbqAaeqaaaqaaiabdMgaPjabg2da9iabigdaXaqaaiabdchaWbqdcqGHris5aOGaeyypa0JaemyBa0gaaa@38BA@. Then, the kernel matrix can be written as

K=(K11K12⋯K1pK21K22⋯K2p⋮⋮⋱⋮Kp1Kp2⋯Kpp)     (8)
 MathType@MTEF@5@5@+=feaafiart1ev1aaatCvAUfKttLearuWrP9MDH5MBPbIqV92AaeXatLxBI9gBaebbnrfifHhDYfgasaacH8akY=wiFfYdH8Gipec8Eeeu0xXdbba9frFj0=OqFfea0dXdd9vqai=hGuQ8kuc9pgc9s8qqaq=dirpe0xb9q8qiLsFr0=vr0=vr0dc8meaabaqaciaacaGaaeqabaqabeGadaaakeaacqWGlbWscqGH9aqpdaqadaqaauaabeqaeqaaaaaabaGaem4saS0aaSbaaSqaaiabigdaXiabigdaXaqabaaakeaacqWGlbWsdaWgaaWcbaGaeGymaeJaeGOmaidabeaaaOqaaiabl+UimbqaaiabdUealnaaBaaaleaacqaIXaqmcqWGWbaCaeqaaaGcbaGaem4saS0aaSbaaSqaaiabikdaYiabigdaXaqabaaakeaacqWGlbWsdaWgaaWcbaGaeGOmaiJaeGOmaidabeaaaOqaaiabl+UimbqaaiabdUealnaaBaaaleaacqaIYaGmcqWGWbaCaeqaaaGcbaGaeSO7I0eabaGaeSO7I0eabaGaeSy8I8eabaGaeSO7I0eabaGaem4saS0aaSbaaSqaaiabdchaWjabigdaXaqabaaakeaacqWGlbWsdaWgaaWcbaGaemiCaaNaeGOmaidabeaaaOqaaiabl+UimbqaaiabdUealnaaBaaaleaacqWGWbaCcqWGWbaCaeqaaaaaaOGaayjkaiaawMcaaiaaxMaacaWLjaWaaeWaaeaacqaI4aaoaiaawIcacaGLPaaaaaa@618B@

where the submatrix *k*_*ij *_is of size *m*_*i *_× *m*_*j*_, and *K*_*ii *_represents the kernel matrix corresponding to the data in the *i*-th class. The class separability of the *i*-th and *j*-th class, denoted by *J*^*ij *^(*i,j *= 1, 2,...,*p*, *i *≠ *j*), is calculated as

Jij(α)=JiijJ2ij=1mi+mjTBij1mi+mj1mi+mjTWij1mi+mj     (9)
 MathType@MTEF@5@5@+=feaafiart1ev1aaatCvAUfKttLearuWrP9MDH5MBPbIqV92AaeXatLxBI9gBaebbnrfifHhDYfgasaacH8akY=wiFfYdH8Gipec8Eeeu0xXdbba9frFj0=OqFfea0dXdd9vqai=hGuQ8kuc9pgc9s8qqaq=dirpe0xb9q8qiLsFr0=vr0=vr0dc8meaabaqaciaacaGaaeqabaqabeGadaaakeaacqWGkbGsdaahaaWcbeqaaiabdMgaPjabdQgaQbaakiabcIcaOGGaciab=f7aHjabcMcaPiabg2da9maalaaabaGaemOsaO0aa0baaSqaaiabdMgaPbqaaiabdMgaPjabdQgaQbaaaOqaaiabdQeaknaaDaaaleaacqaIYaGmaeaacqWGPbqAcqWGQbGAaaaaaOGaeyypa0ZaaSaaaeaacqaIXaqmdaqhaaWcbaGaemyBa02aaSbaaWqaaiabdMgaPbqabaWccqGHRaWkcqWGTbqBdaWgaaadbaGaemOAaOgabeaaaSqaaiabdsfaubaakiabdkeacnaaCaaaleqabaGaemyAaKMaemOAaOgaaOGaeGymaeZaaSbaaSqaaiabd2gaTnaaBaaameaacqWGPbqAaeqaaSGaey4kaSIaemyBa02aaSbaaWqaaiabdQgaQbqabaaaleqaaaGcbaGaeGymaeZaa0baaSqaaiabd2gaTnaaBaaameaacqWGPbqAaeqaaSGaey4kaSIaemyBa02aaSbaaWqaaiabdQgaQbqabaaaleaacqWGubavaaGccqWGxbWvdaahaaWcbeqaaiabdMgaPjabdQgaQbaakiabigdaXmaaBaaaleaacqWGTbqBdaWgaaadbaGaemyAaKgabeaaliabgUcaRiabd2gaTnaaBaaameaacqWGQbGAaeqaaaWcbeaaaaGccaWLjaGaaCzcamaabmaabaGaeGyoaKdacaGLOaGaayzkaaaaaa@6ECC@

where the between-class and within-class kernel scatter matrices *B*^*ij *^and *W*^*ij *^are defined as

Bij=(1miKii001mjKjj)−1mi+mj(KiiKijKjiKjj)
 MathType@MTEF@5@5@+=feaafiart1ev1aaatCvAUfKttLearuWrP9MDH5MBPbIqV92AaeXatLxBI9gBaebbnrfifHhDYfgasaacH8akY=wiFfYdH8Gipec8Eeeu0xXdbba9frFj0=OqFfea0dXdd9vqai=hGuQ8kuc9pgc9s8qqaq=dirpe0xb9q8qiLsFr0=vr0=vr0dc8meaabaqaciaacaGaaeqabaqabeGadaaakeaacqWGcbGqdaahaaWcbeqaaiabdMgaPjabdQgaQbaakiabg2da9maabmaabaqbaeqabiGaaaqaamaalaaabaGaeGymaedabaGaemyBa02aaSbaaSqaaiabdMgaPbqabaaaaOGaem4saS0aaSbaaSqaaiabdMgaPjabdMgaPbqabaaakeaacqaIWaamaeaacqaIWaamaeaadaWcaaqaaiabigdaXaqaaiabd2gaTnaaBaaaleaacqWGQbGAaeqaaaaakiabdUealnaaBaaaleaacqWGQbGAcqWGQbGAaeqaaaaaaOGaayjkaiaawMcaaiabgkHiTmaalaaabaGaeGymaedabaGaemyBa02aaSbaaSqaaiabdMgaPbqabaGccqGHRaWkcqWGTbqBdaWgaaWcbaGaemOAaOgabeaaaaGcdaqadaqaauaabeqaciaaaeaacqWGlbWsdaWgaaWcbaGaemyAaKMaemyAaKgabeaaaOqaaiabdUealnaaBaaaleaacqWGPbqAcqWGQbGAaeqaaaGcbaGaem4saS0aaSbaaSqaaiabdQgaQjabdMgaPbqabaaakeaacqWGlbWsdaWgaaWcbaGaemOAaOMaemOAaOgabeaaaaaakiaawIcacaGLPaaaaaa@5FAD@

Wij=Dij−(1miKii001mjKjj)
 MathType@MTEF@5@5@+=feaafiart1ev1aaatCvAUfKttLearuWrP9MDH5MBPbIqV92AaeXatLxBI9gBaebbnrfifHhDYfgasaacH8akY=wiFfYdH8Gipec8Eeeu0xXdbba9frFj0=OqFfea0dXdd9vqai=hGuQ8kuc9pgc9s8qqaq=dirpe0xb9q8qiLsFr0=vr0=vr0dc8meaabaqaciaacaGaaeqabaqabeGadaaakeaacqWGxbWvdaahaaWcbeqaaiabdMgaPjabdQgaQbaakiabg2da9iabdseaenaaCaaaleqabaGaemyAaKMaemOAaOgaaOGaeyOeI0YaaeWaaeaafaqabeGacaaabaWaaSaaaeaacqaIXaqmaeaacqWGTbqBdaWgaaWcbaGaemyAaKgabeaaaaGccqWGlbWsdaWgaaWcbaGaemyAaKMaemyAaKgabeaaaOqaaiabicdaWaqaaiabicdaWaqaamaalaaabaGaeGymaedabaGaemyBa02aaSbaaSqaaiabdQgaQbqabaaaaOGaem4saS0aaSbaaSqaaiabdQgaQjabdQgaQbqabaaaaaGccaGLOaGaayzkaaaaaa@4A3E@

in which *D*^*ij *^denotes a diagonal matrix whose diagonal elements are composed of the diagonal entries of the matrix *K*_*ii *_and *K*_*jj*_. We also denote the between-class and within-class kernel matrices corresponding to the basic kernel by B0ij
 MathType@MTEF@5@5@+=feaafiart1ev1aaatCvAUfKttLearuWrP9MDH5MBPbIqV92AaeXatLxBI9gBaebbnrfifHhDYfgasaacH8akY=wiFfYdH8Gipec8Eeeu0xXdbba9frFj0=OqFfea0dXdd9vqai=hGuQ8kuc9pgc9s8qqaq=dirpe0xb9q8qiLsFr0=vr0=vr0dc8meaabaqaciaacaGaaeqabaqabeGadaaakeaacqWGcbGqdaqhaaWcbaGaeGimaadabaGaemyAaKMaemOAaOgaaaaa@318C@ and W0ij
 MathType@MTEF@5@5@+=feaafiart1ev1aaatCvAUfKttLearuWrP9MDH5MBPbIqV92AaeXatLxBI9gBaebbnrfifHhDYfgasaacH8akY=wiFfYdH8Gipec8Eeeu0xXdbba9frFj0=OqFfea0dXdd9vqai=hGuQ8kuc9pgc9s8qqaq=dirpe0xb9q8qiLsFr0=vr0=vr0dc8meaabaqaciaacaGaaeqabaqabeGadaaakeaacqWGxbWvdaqhaaWcbaGaeGimaadabaGaemyAaKMaemOAaOgaaaaa@31B6@ respectively.

In each iteration of the updating algorithm, we first find the class index (*u, v*) that corresponds to the minimum *J*^*ij *^in current step, then the value of *α *is updated in such a way that the class separability of the *u*-th and *v*-th class *J*^*uv *^will be maximized. In other words, the objective of the kernel optimization becomes



It is easy to modify the kernel optimization algorithm from the case of binary class data to the case of multi-class data. The detailed kernel optimization algorithm for multi-class data set is summarized below, where Γ_*ij *_denotes the union of the data index sets of the *i*-th and *j*-th class, and *q*(Γ_*ij*_) and *K*_1_(Γ_*ij*_,:) represent the submatrix extraction as in MATLAB.

1. Group the data according to their class labels. Calculate *k*_0 _and *K*_1_.

2. Initialize *α*^(0) ^by a vector (1,0,..., 0)^*T*^, and set *n *= 0.

3. Calculate *q *= *K*_1_*α*^(*n*)^, J1ij
 MathType@MTEF@5@5@+=feaafiart1ev1aaatCvAUfKttLearuWrP9MDH5MBPbIqV92AaeXatLxBI9gBaebbnrfifHhDYfgasaacH8akY=wiFfYdH8Gipec8Eeeu0xXdbba9frFj0=OqFfea0dXdd9vqai=hGuQ8kuc9pgc9s8qqaq=dirpe0xb9q8qiLsFr0=vr0=vr0dc8meaabaqaciaacaGaaeqabaqabeGadaaakeaacqWGkbGsdaqhaaWcbaGaeGymaedabaGaemyAaKMaemOAaOgaaaaa@319E@ = *q*(Γ_*ij*_)^*T *^B0ij
 MathType@MTEF@5@5@+=feaafiart1ev1aaatCvAUfKttLearuWrP9MDH5MBPbIqV92AaeXatLxBI9gBaebbnrfifHhDYfgasaacH8akY=wiFfYdH8Gipec8Eeeu0xXdbba9frFj0=OqFfea0dXdd9vqai=hGuQ8kuc9pgc9s8qqaq=dirpe0xb9q8qiLsFr0=vr0=vr0dc8meaabaqaciaacaGaaeqabaqabeGadaaakeaacqWGcbGqdaqhaaWcbaGaeGimaadabaGaemyAaKMaemOAaOgaaaaa@318C@*q*(Γ_*ij*_), J2ij
 MathType@MTEF@5@5@+=feaafiart1ev1aaatCvAUfKttLearuWrP9MDH5MBPbIqV92AaeXatLxBI9gBaebbnrfifHhDYfgasaacH8akY=wiFfYdH8Gipec8Eeeu0xXdbba9frFj0=OqFfea0dXdd9vqai=hGuQ8kuc9pgc9s8qqaq=dirpe0xb9q8qiLsFr0=vr0=vr0dc8meaabaqaciaacaGaaeqabaqabeGadaaakeaacqWGkbGsdaqhaaWcbaGaeGOmaidabaGaemyAaKMaemOAaOgaaaaa@31A0@ = *q*(Γ_*ij*_)^*T *^W0ij
 MathType@MTEF@5@5@+=feaafiart1ev1aaatCvAUfKttLearuWrP9MDH5MBPbIqV92AaeXatLxBI9gBaebbnrfifHhDYfgasaacH8akY=wiFfYdH8Gipec8Eeeu0xXdbba9frFj0=OqFfea0dXdd9vqai=hGuQ8kuc9pgc9s8qqaq=dirpe0xb9q8qiLsFr0=vr0=vr0dc8meaabaqaciaacaGaaeqabaqabeGadaaakeaacqWGxbWvdaqhaaWcbaGaeGimaadabaGaemyAaKMaemOAaOgaaaaa@31B6@*q*(Γ_*ij*_), and *J*^*ij*^, where *i*, *j *= l,2,...,*p*, and *i *≠ *j*.

4. Find (u,v)=arg⁡min⁡ij
 MathType@MTEF@5@5@+=feaafiart1ev1aaatCvAUfKttLearuWrP9MDH5MBPbIqV92AaeXatLxBI9gBaebbnrfifHhDYfgasaacH8akY=wiFfYdH8Gipec8Eeeu0xXdbba9frFj0=OqFfea0dXdd9vqai=hGuQ8kuc9pgc9s8qqaq=dirpe0xb9q8qiLsFr0=vr0=vr0dc8meaabaqaciaacaGaaeqabaqabeGadaaakeaacqGGOaakcqWG1bqDcqGGSaalcqWG2bGDcqGGPaqkcqGH9aqpcyGGHbqycqGGYbGCcqGGNbWzdaWfqaqaaiGbc2gaTjabcMgaPjabc6gaUbWcbaGaemyAaKMaemOAaOgabeaaaaa@3E4D@*J*^*ij*^(*α*), and calculate M0uv
 MathType@MTEF@5@5@+=feaafiart1ev1aaatCvAUfKttLearuWrP9MDH5MBPbIqV92AaeXatLxBI9gBaebbnrfifHhDYfgasaacH8akY=wiFfYdH8Gipec8Eeeu0xXdbba9frFj0=OqFfea0dXdd9vqai=hGuQ8kuc9pgc9s8qqaq=dirpe0xb9q8qiLsFr0=vr0=vr0dc8meaabaqaciaacaGaaeqabaqabeGadaaakeaacqWGnbqtdaqhaaWcbaGaeGimaadabaGaemyDauNaemODayhaaaaa@31D2@ = *K*_1_(Γ_*uv*_,:)^*T *^B0uv
 MathType@MTEF@5@5@+=feaafiart1ev1aaatCvAUfKttLearuWrP9MDH5MBPbIqV92AaeXatLxBI9gBaebbnrfifHhDYfgasaacH8akY=wiFfYdH8Gipec8Eeeu0xXdbba9frFj0=OqFfea0dXdd9vqai=hGuQ8kuc9pgc9s8qqaq=dirpe0xb9q8qiLsFr0=vr0=vr0dc8meaabaqaciaacaGaaeqabaqabeGadaaakeaacqWGcbGqdaqhaaWcbaGaeGimaadabaGaemyDauNaemODayhaaaaa@31BC@*K*_1_(Γ_*uv*_,:), and N0uv
 MathType@MTEF@5@5@+=feaafiart1ev1aaatCvAUfKttLearuWrP9MDH5MBPbIqV92AaeXatLxBI9gBaebbnrfifHhDYfgasaacH8akY=wiFfYdH8Gipec8Eeeu0xXdbba9frFj0=OqFfea0dXdd9vqai=hGuQ8kuc9pgc9s8qqaq=dirpe0xb9q8qiLsFr0=vr0=vr0dc8meaabaqaciaacaGaaeqabaqabeGadaaakeaacqWGobGtdaqhaaWcbaGaeGimaadabaGaemyDauNaemODayhaaaaa@31D4@ = *K*_1_(Γ_*uv*_,:)^*T *^W0uv
 MathType@MTEF@5@5@+=feaafiart1ev1aaatCvAUfKttLearuWrP9MDH5MBPbIqV92AaeXatLxBI9gBaebbnrfifHhDYfgasaacH8akY=wiFfYdH8Gipec8Eeeu0xXdbba9frFj0=OqFfea0dXdd9vqai=hGuQ8kuc9pgc9s8qqaq=dirpe0xb9q8qiLsFr0=vr0=vr0dc8meaabaqaciaacaGaaeqabaqabeGadaaakeaacqWGxbWvdaqhaaWcbaGaeGimaadabaGaemyDauNaemODayhaaaaa@31E6@*K*_1_(Γ_*uv*_,:).

5. Update *α*^(*n*)^

α(n+1)=α(n)+η(n)(1J2uvM0uv−JuvJ2uvN0uv)α(n)
 MathType@MTEF@5@5@+=feaafiart1ev1aaatCvAUfKttLearuWrP9MDH5MBPbIqV92AaeXatLxBI9gBaebbnrfifHhDYfgasaacH8akY=wiFfYdH8Gipec8Eeeu0xXdbba9frFj0=OqFfea0dXdd9vqai=hGuQ8kuc9pgc9s8qqaq=dirpe0xb9q8qiLsFr0=vr0=vr0dc8meaabaqaciaacaGaaeqabaqabeGadaaakeaaiiGacqWFXoqydaahaaWcbeqaaiabcIcaOiabd6gaUjabgUcaRiabigdaXiabcMcaPaaakiabg2da9iab=f7aHnaaCaaaleqabaGaeiikaGIaemOBa4MaeiykaKcaaOGaey4kaSIae83TdGMaeiikaGIaemOBa4MaeiykaKIaeiikaGYaaSaaaeaacqaIXaqmaeaacqWGkbGsdaqhaaWcbaGaeGOmaidabaGaemyDauNaemODayhaaaaakiabd2eannaaDaaaleaacqaIWaamaeaacqWG1bqDcqWG2bGDaaGccqGHsisldaWcaaqaaiabdQeaknaaCaaaleqabaGaemyDauNaemODayhaaaGcbaGaemOsaO0aa0baaSqaaiabikdaYaqaaiabdwha1jabdAha2baaaaGccqWGobGtdaqhaaWcbaGaeGimaadabaGaemyDauNaemODayhaaOGaeiykaKIae8xSde2aaWbaaSqabeaacqGGOaakcqWGUbGBcqGGPaqkaaaaaa@6087@

and normalize *α*^(*n*+1) ^so that ||*α*^(*n*+1) ^|| = 1.

6. If *n *reaches a prespecified number *N*, stop. Otherwise, set *n *= *n *+ 1, go to step 3.

### 0.4 KNN classification using the optimized kernel distance metric

Given two samples *x,y *∈ **R**^*d*^, the inner product is defined as: *x*·*y *= <*x*, *y *> = *k*(*x*, *y*); therefore, their derived distance can be calculated

*d*(*x*, *y*) = <*x*, *x *> + <*y*, *y *> -2 <*x, y *> = *k*(*x*, *x*) + *k*(*y*, *y*) - 2*k*(*x*, *y*).

Using our data-dependent kernel model, the distance can be expressed as

*d*(*x*, *y*) = *q*^2^(*x*) + *q*^2^(*y*) - 2*q*(*x*)*q*(*y*)*k*_0_(*x*, *y*) = [*q*(*x*) - *q*(*y*)]^2 ^+ 2*q*(*x*)*q*(*y*)(1 - *k*_0_(*x*, *y*))

where we assume that the basic kernel function satisfy: *k*_0_(*x,x*) = 1, just like the Gaussian function.

Since the kernel optimization scheme increases the class separability of the data in the feature space, the performances of kernel machines should be improved. However, for the classification of gene expression data, due to the small size of training samples, the kernel optimization, which performs on training data, may cause overfitting, which means the algorithm may work very well on the training data, but worse on the test data. To handle this problem, we adopted a disturbed resampling strategy to increase the sample size of the training data.

Suppose that {*x*_*i*_, *ζ*_*i*_} (*i *= 1,2, ... *m*) are the training data, we construct a new set of training data {*y*_*i*_*,ξ*_*i*_}(*i *= 1,2,...,3*m*), where

yi={xiif 1≤i≤mxr+εif i>m     (10)
 MathType@MTEF@5@5@+=feaafiart1ev1aaatCvAUfKttLearuWrP9MDH5MBPbIqV92AaeXatLxBI9gBaebbnrfifHhDYfgasaacH8akY=wiFfYdH8Gipec8Eeeu0xXdbba9frFj0=OqFfea0dXdd9vqai=hGuQ8kuc9pgc9s8qqaq=dirpe0xb9q8qiLsFr0=vr0=vr0dc8meaabaqaciaacaGaaeqabaqabeGadaaakeaacqWG5bqEdaWgaaWcbaGaemyAaKgabeaakiabg2da9maaceaabaqbaeaabiGaaaqaaiabdIha4naaBaaaleaacqWGPbqAaeqaaaGcbaGaeeyAaKMaeeOzayMaeeiiaaIaeGymaeJaeyizImQaemyAaKMaeyizImQaemyBa0gabaGaemiEaG3aaSbaaSqaaiabdkhaYbqabaGccqGHRaWkiiGacqWF1oqzaeaacqqGPbqAcqqGMbGzcqqGGaaicqWGPbqAcqGH+aGpcqWGTbqBaaaacaGL7baacaWLjaGaaCzcamaabmaabaGaeGymaeJaeGimaadacaGLOaGaayzkaaaaaa@510C@

in which *x*_*r *_is a sample randomly selected form {*x*_*i*_} with replacement and *ε *denotes a normal random disturb, that is, *ε *~*N*(0, σε2
 MathType@MTEF@5@5@+=feaafiart1ev1aaatCvAUfKttLearuWrP9MDH5MBPbIqV92AaeXatLxBI9gBaebbnrfifHhDYfgasaacH8akY=wiFfYdH8Gipec8Eeeu0xXdbba9frFj0=OqFfea0dXdd9vqai=hGuQ8kuc9pgc9s8qqaq=dirpe0xb9q8qiLsFr0=vr0=vr0dc8meaabaqaciaacaGaaeqabaqabeGadaaakeaaiiGacqWFdpWCdaqhaaWcbaGae8xTdugabaGaeGOmaidaaaaa@3137@). The class labels are determined as

ξi={ζiif 1≤i≤mζrif i>m
 MathType@MTEF@5@5@+=feaafiart1ev1aaatCvAUfKttLearuWrP9MDH5MBPbIqV92AaeXatLxBI9gBaebbnrfifHhDYfgasaacH8akY=wiFfYdH8Gipec8Eeeu0xXdbba9frFj0=OqFfea0dXdd9vqai=hGuQ8kuc9pgc9s8qqaq=dirpe0xb9q8qiLsFr0=vr0=vr0dc8meaabaqaciaacaGaaeqabaqabeGadaaakeaaiiGacqWF+oaEdaWgaaWcbaGaemyAaKgabeaakiabg2da9maaceaabaqbaeaabiGaaaqaaiab=z7a6naaBaaaleaacqWGPbqAaeqaaaGcbaGaeeyAaKMaeeOzayMaeeiiaaIaeGymaeJaeyizImQaemyAaKMaeyizImQaemyBa0gabaGae8NTdO3aaSbaaSqaaiabdkhaYbqabaaakeaacqqGPbqAcqqGMbGzcqqGGaaicqWGPbqAcqGH+aGpcqWGTbqBaaaacaGL7baaaaa@4A9E@

Due to the very high dimensionality and small number of the patient samples, the training data are sparsely distributed in the high dimensional Euclidean space. It is reasonable to assume that the near points of a training datum have the same class characteristic as that of the training datum. Experimentally, using the technique of disturbed resampling (Eq.(l0)), we can effectively reduce the possible overfitting and computational instability, which are mainly caused by the lack of enough training samples for the gene expression data.

## Abbreviations

KNN: K-nearest-Neighbor

SVM: support vector machine

DLDA: diagonal linear discriminant analysis

ULDA: uncorrelated linear discriminant analysis

KerNN: kernel optimization-based KNN

ALL: acute lymphoblastic leukemia

AML: acute myeloid leukemia

MLL: mixed lineage leukemia

CNS: embryonal tumor of central nervous system

## Authors' contributions

HX and XWC conceived the study. HX designed and implemented the algorithms, and drafted the manuscript. XWC coordinated the study, participated in the algorithm design, and helped draft the manuscript.

## Availability

The core source codes of our algorithms are available at 

## Figures and Tables

**Table 1:** Comparison of the classifiers in terms of the best results. The comparison of all the classifiers in terms of the best results of the average test error rates (%). For each data set, we chose the *N*_*f *_most discriminatory genes, where *N*_*f *_= 10, 20, 40, 60, 80, 100, 200, 400, 600, 800, 1000, 1200, 1400, 1600, 1800, 2000, respectively; repeated the experiment 100 times at each value of *N*_*f*_; and then, calculated the average test error rates and their standard deviations over the 100 experiments. In comparison, we assign a classifier a score 1 as it achieves the best result on one data set, and 2 if it achieves the next best result, and so on. The average score roughly evaluates the global performance of a classifier on these twelve data sets.

	KNN	ULDA	DLDA	SVM	KerNN
*ALL-AML*	3.32 (1.21)	3.08 (1.09)	2.95 (0.78)	**2.70 **(0.00)	**2.70 **(0.00)
*ALL-MLL-AML*	6.17 (2.75)	**2.14 **(1.97)	5.19 (2.95)	2.83 (2.37)	3.21 (2.18)
*CNS*	19.52 (5.88)	**12.26 **(7.04)	22.42 (5.58)	13.35 (7.52)	15.32 (5.60)
*Breast-ER*	7.12 (4.12)	4.92 (4.40)	**3.21 **(3.04)	4.64 (4.39)	4.48 (4.45)
*Breast-LN*	13.12 (5.91)	9.92 (5.16)	**7.76 **(4.85)	7.92 (5.39)	8.36 (4.48)
*Colon*	14.03 (3.76)	16.84 (6.14)	12.65 (4.58)	11.84 (4.28)	**11.58 **(4.97)
*Lung*	1.21 (0.98)	0.81 (0.73)	0.47 (0.57)	0.53 (0.61)	**0.31 **(0.54)
*Lymphoma*	2.05 (2.58)	2.05 (2.09)	6.23 (2.88)	**1.03 **(1.59)	1.90 (2.05)
*Ovarian*	0.74 (0.87)	0.02 (0.13)	1.58 (0.81)	0.17 (0.42)	**0.01 **(0.08)
*Prostate*	7.41 (2.47)	5.22 (2.99)	6.73 (3.02)	**4.86 **(2.77)	4.90 (2.53)
*Subtypes*	2.57 (0.86)	**1.73 **(0.90)	2.45 (0.92)	2.60 (1.02)	2.42 (0.82)

Average Score	4.5	2.8	3.3	2.3	**1.9**
